# 1-Benzyl-3-[(4-methyl­phen­yl)imino]­indolin-2-one

**DOI:** 10.1107/S1600536812024506

**Published:** 2012-06-13

**Authors:** Adebomi A. Ikotun, Pius O. Adelani, Gabriel O. Egharevba

**Affiliations:** a Chemistry and Industrial Chemistry Department, Bowen University, Iwo, Nigeria; b Department of Civil Engineering and Geological Sciences and Department of Chemistry and Biochemistry, University of Notre Dame, Notre Dame, Indiana 46556, USA; c Chemistry Department, Obafemi Awolowo University, Ile-ife, Nigeria

## Abstract

In the title compound, C_22_H_18_N_2_O, the phenyl and tolyl rings make dihedral angles of 84.71 (7) and 65.11 (6)°, respectively, with the isatin group. The aromatic rings make a dihedral angle of 60.90 (8)°. The imino C=N double bond, exists in an *E* conformation. In the crystal, mol­ecules are linked by weak π–π stacking inter­actions [centroid–centroid distance = 3.6598 (13) Å].

## Related literature

For background to isatin, its derivatives and their biological significance, see: Chazeau *et al.* (1992 ▶); Igosheva *et al.* (2004 ▶); Medvedev *et al.* (1996 ▶); Abele *et al.* (2003 ▶). For metal complexes of isatin derivatives and their biological significance, see: Rodriguez-Arguelles *et al.* (2004 ▶); Singh *et al.* (2005 ▶); Chohan *et al.* (2006 ▶); Adetoye *et al.* (2009 ▶); Ikotun *et al.* (2012 ▶). For *N*-benzyl isatin, its derivatives and biological significance, see Akkurt *et al.* (2006 ▶); Jarrahpour & Khalili (2007 ▶); Cao *et al.* (2009 ▶).
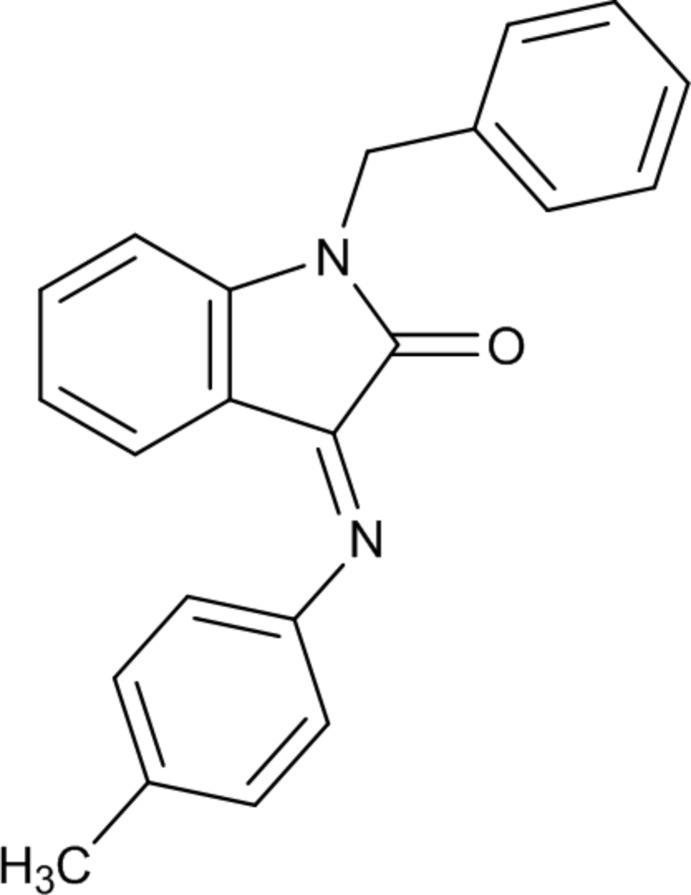


## Experimental

### 

#### Crystal data

C_22_H_18_N_2_O
*M* *_r_* = 326.38
Monoclinic, 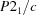

*a* = 10.174 (2) Å
*b* = 15.086 (4) Å
*c* = 11.714 (3) Å
β = 113.596 (3)°
*V* = 1647.5 (7) Å^3^
*Z* = 4
Mo *K*α radiation
μ = 0.08 mm^−1^
*T* = 296 K
0.04 × 0.02 × 0.01 mm

#### Data collection

Bruker SMART CCD area-detector diffractometer
18815 measured reflections
3763 independent reflections
2456 reflections with *I* > 2σ(*I*)
*R*_int_ = 0.067

#### Refinement

*R*[*F* ^2^ > 2σ(*F* ^2^)] = 0.042
*wR*(*F* ^2^) = 0.105
*S* = 0.92
3763 reflections
227 parameters
H-atom parameters constrained
Δρ_max_ = 0.35 e Å^−3^
Δρ_min_ = −0.31 e Å^−3^

### 

Data collection: *SMART* (Bruker, 2000 ▶); cell refinement: *SAINT* (Bruker, 2000 ▶); data reduction: *SAINT*; program(s) used to solve structure: *SHELXS97* (Sheldrick, 2008 ▶); program(s) used to refine structure: *SHELXL97* (Sheldrick, 2008 ▶); molecular graphics: *PLATON* (Spek, 2009 ▶); software used to prepare material for publication: *SHELXTL* (Sheldrick, 2008 ▶).

## Supplementary Material

Crystal structure: contains datablock(s) I, global. DOI: 10.1107/S1600536812024506/bx2411sup1.cif

Structure factors: contains datablock(s) I. DOI: 10.1107/S1600536812024506/bx2411Isup2.hkl

Supplementary material file. DOI: 10.1107/S1600536812024506/bx2411Isup3.cml

Additional supplementary materials:  crystallographic information; 3D view; checkCIF report

## Figures and Tables

**Fig. 1.**
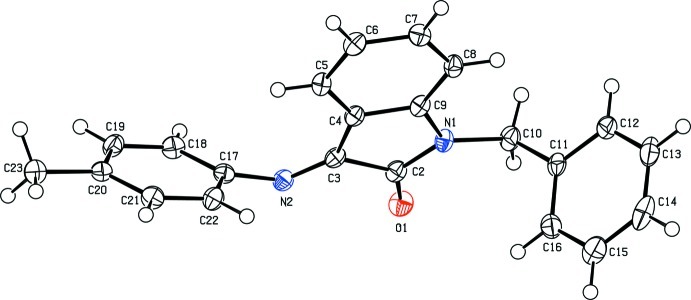
The molecular structure of the title compound showing the labelled atoms; thermal ellipsoid are drawn at 50% probability level.

**Fig. 2.**
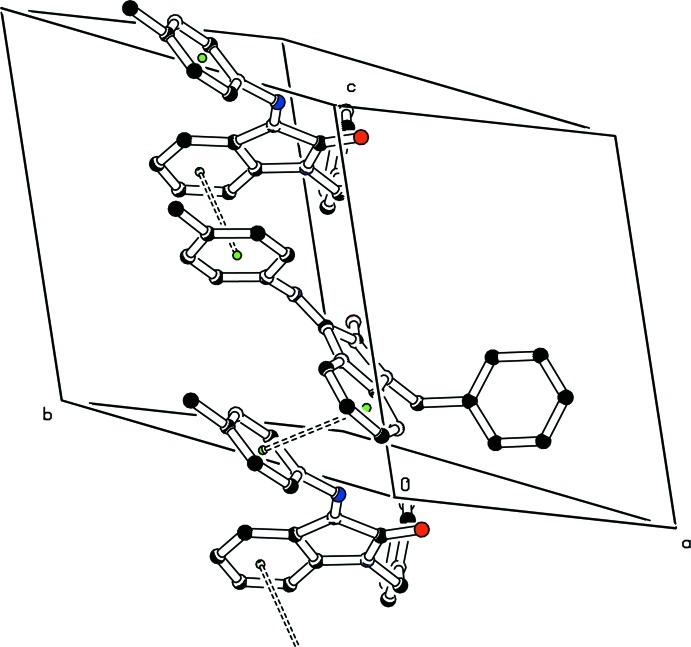
Part of the crystal structure showing π—π stacking interaction (centroid-centroid distance 3.6598 (13) Å (Cg1=C4/C5/C6/C7/C8/C9 ; Cg2i=C17/C18/C19/C20/C21/C22, symmetry code (i): x,1/2-y, 1/2+z).

## supplementary crystallographic information

### Comment

Indole-2, 3-dione commonly known as isatin is an endogenous indole present in mammalian tissues and fluids (Igosheva *et al.*, 2004). It has largely been used as a versatile reagent in organic synthesis, to obtain heterocyclic compounds, and as a raw material for drugs (Abele *et al.*, 2003). Several novel Schiff bases of isatin have been reported with a variety of pharmacological actions, including anticonvulsant, antimicrobial and antiviral activities, inhibition of monoamine oxidase (Medvedev *et al.*, 1996). The study of the metal complexes of the Schiff base ligands derived from isatin and their biological applications has also received much attention (Singh *et al.*, 2005; Chohan *et al.*, 2006; Ikotun *et al.*, 2012). Some first row transition metal complexes of the Schiff base of isatin derivatives were designed, prepared and characterized by spectroscopic means (Adetoye *et al.*, 2009). The significance of these metal complexes of isatin derivatives has even been extended to the design of novel anticancer drugs (Rodriguez-Arguelles *et al.*, 2004). *N*-benzylindole-2, 3-dione (*N*-benzylisatin) has also been prepared and the X-ray crystallographic structure has been established (Akkurt *et al.*, 2006). *N*-alkylated isatins have interesting pharmacological activities such as antibacterial and anticancer (Chazeau *et al.*, 1992). They are also reversible and competitive inhibitors of monoamine oxidase A and B (Medvedev *et al.*, 1996). Some mono- and bis-spiro-b- benzylisatin have been prepared and characterized by spectroscopic means (Jarrahpour *et al.*, 2007). A series of *N*-benzyl isatin oximes have also been developed as inhibitors of the mitogen-activated kinase, KNK3 (Cao *et al.*, 2009). Thus the motivation and need to design novel Schiff bases of *N*-benzyl isatin, which would be of great biological significance, is the propelling force for this research. In the title compound , C_22_H_18_N_2_O, Fig. 1, the phenyl and benzene rings make dihedral angles of 84.71 (7)° and 65.11 (6)° with isatin group respectively. The aromatic rings make a dihedral angle of 60.90 (8)°.The imino C═N double bond, exists in an E conformation. In the crystal the molecules are linked by weak π—π stacking interaction (centroid-centroid distance 3.6598 (13) Å (Cg1=C4/C5/C6/C7/C8/C9 ; Cg2^i^=C17/C18/C19/C20/C21/C22, symmetry code (i): x,1/2-y, 1/2+z), Fig. 2.

### Experimental

*N*-benzylisatin was first prepared and recrystallized in ethanol using the method of Akkurt *et al.*, 2006 with slight modifications. *N*-benzylisatin (2.00 g; 8.44 mmol) was then dissolved in 30 ml hot ethanol. P-toluidine (0.90 g; 8.44 mmol) was dissolved in 10 ml ethanol. The solutions were mixed and refluxed for 6 h. The solution was allowed to cool and the deep orange solid was filtered under vacuum. The product was purified with flash column chromatography and the orange crystal as analyzed. The product was obtained at a yield of 78% (2.13 g). Flash Column Chromatographic purification of the product was carried out using a mixture of chloroform: diethyl ether (50%:50%) and single X-ray suitable crystals were got after the solvent was evaporated under vacuum.

### Refinement

The H atoms of the water molecule were located on a Fourier difference map, restrained by *DFIX* command 0.85 Å for O— H distances and by *DFIX* 1.39 Å for H···H distance, and refined as riding with *U*_iso_(H) = 1.5U_eq_(O). Other atoms were placed in their calculated positions, with C—H = 0.93 or 0.96 Å, and refined using a riding model, with *U*_iso_(H) = 1.2 or 1.5U_eq_(C).

### Crystal data

**Table:**

C_22_H_18_N_2_O	*F*(000) = 688
*M**_r_* = 326.38	*D*_x_ = 1.316 Mg m^−^^3^
Monoclinic, *P*2_1_/*c*	Melting point: 427 K
Hall symbol: -P 2ybc	Mo *K*α radiation, λ = 0.71073 Å
*a* = 10.174 (2) Å	Cell parameters from 3069 reflections
*b* = 15.086 (4) Å	θ = 2.6–25.6°
*c* = 11.714 (3) Å	µ = 0.08 mm^−^^1^
β = 113.596 (3)°	*T* = 296 K
*V* = 1647.5 (7) Å^3^	Rectangular plate, orange
*Z* = 4	0.04 × 0.02 × 0.01 mm

### Data collection

**Table:**

Bruker SMART CCD area-detector diffractometer	2456 reflections with *I* > 2σ(*I*)
Radiation source: fine-focus sealed tube	*R*_int_ = 0.067
Graphite monochromator	θ_max_ = 27.5°, θ_min_ = 2.2°
phi and ω scans	*h* = −12→13
18815 measured reflections	*k* = −19→19
3763 independent reflections	*l* = −15→15

### Refinement

**Table:**

Refinement on *F*^2^	Secondary atom site location: difference Fourier map
Least-squares matrix: full	Hydrogen site location: inferred from neighbouring sites
*R*[*F*^2^ > 2σ(*F*^2^)] = 0.042	H-atom parameters constrained
*wR*(*F*^2^) = 0.105	*w* = 1/[σ^2^(*F*_o_^2^) + (0.0513*P*)^2^] where *P* = (*F*_o_^2^ + 2*F*_c_^2^)/3
*S* = 0.92	(Δ/σ)_max_ < 0.001
3763 reflections	Δρ_max_ = 0.35 e Å^−^^3^
227 parameters	Δρ_min_ = −0.31 e Å^−^^3^
0 restraints	Extinction correction: *SHELXL97* (Sheldrick, 2008), Fc^*^=kFc[1+0.001xFc^2^λ^3^/sin(2θ)]^-1/4^
Primary atom site location: structure-invariant direct methods	Extinction coefficient: 0.0075 (12)

### Special details

**Table:**

Geometry. All e.s.d.'s (except the e.s.d. in the dihedral angle between two l.s. planes) are estimated using the full covariance matrix. The cell e.s.d.'s are taken into account individually in the estimation of e.s.d.'s in distances, angles and torsion angles; correlations between e.s.d.'s in cell parameters are only used when they are defined by crystal symmetry. An approximate (isotropic) treatment of cell e.s.d.'s is used for estimating e.s.d.'s involving l.s. planes.
Refinement. Refinement of *F*^2^ against ALL reflections. The weighted *R*-factor *wR* and goodness of fit *S* are based on *F*^2^, conventional *R*-factors *R* are based on *F*, with *F* set to zero for negative *F*^2^. The threshold expression of *F*^2^ > σ(*F*^2^) is used only for calculating *R*-factors(gt) *etc*. and is not relevant to the choice of reflections for refinement. *R*-factors based on *F*^2^ are statistically about twice as large as those based on *F*, and *R*- factors based on ALL data will be even larger.

### Fractional atomic coordinates and isotropic or equivalent isotropic displacement parameters (Å^2^)

**Table:**

	*x*	*y*	*z*	*U*_iso_*/*U*_eq_	
O1	0.30165 (11)	0.19312 (7)	0.89390 (10)	0.0256 (3)	
N1	0.23619 (13)	0.32278 (8)	0.77977 (12)	0.0192 (3)	
N2	0.06155 (13)	0.22984 (8)	0.95540 (12)	0.0195 (3)	
C18	−0.15861 (16)	0.18456 (10)	0.97075 (14)	0.0221 (4)	
H18A	−0.1610	0.1353	0.9223	0.027*	
C19	−0.26219 (17)	0.19479 (11)	1.01801 (15)	0.0237 (4)	
H19A	−0.3354	0.1531	0.9984	0.028*	
C6	−0.10985 (17)	0.48946 (10)	0.72028 (14)	0.0217 (4)	
H6A	−0.1886	0.5253	0.7085	0.026*	
C8	0.09598 (16)	0.45662 (10)	0.67264 (14)	0.0204 (4)	
H8A	0.1541	0.4694	0.6305	0.024*	
C9	0.12438 (16)	0.38541 (10)	0.75280 (14)	0.0179 (3)	
C11	0.43743 (16)	0.40255 (10)	0.75565 (14)	0.0197 (4)	
C22	−0.04756 (17)	0.32009 (10)	1.06994 (14)	0.0226 (4)	
H22A	0.0236	0.3629	1.0872	0.027*	
C5	−0.08057 (16)	0.41752 (10)	0.80063 (14)	0.0203 (4)	
H5A	−0.1394	0.4046	0.8421	0.024*	
C10	0.34082 (17)	0.32210 (10)	0.72300 (15)	0.0233 (4)	
H10A	0.2900	0.3190	0.6332	0.028*	
H10B	0.3996	0.2693	0.7499	0.028*	
C17	−0.05075 (16)	0.24753 (10)	0.99516 (14)	0.0192 (3)	
C4	0.03861 (16)	0.36512 (10)	0.81794 (14)	0.0178 (3)	
C20	−0.25950 (16)	0.26584 (10)	1.09418 (14)	0.0211 (4)	
C7	−0.02307 (16)	0.50861 (10)	0.65726 (14)	0.0209 (4)	
H7A	−0.0449	0.5571	0.6038	0.025*	
C21	−0.15100 (17)	0.32812 (10)	1.11853 (15)	0.0236 (4)	
H21A	−0.1476	0.3765	1.1688	0.028*	
C3	0.09602 (16)	0.28278 (10)	0.88691 (14)	0.0182 (3)	
C2	0.22451 (16)	0.25759 (10)	0.85647 (14)	0.0196 (3)	
C16	0.52131 (17)	0.42307 (11)	0.87874 (15)	0.0249 (4)	
H16A	0.5174	0.3873	0.9420	0.030*	
C12	0.44522 (17)	0.45665 (12)	0.66264 (16)	0.0289 (4)	
H12A	0.3903	0.4435	0.5795	0.035*	
C13	0.53422 (18)	0.53003 (12)	0.69284 (18)	0.0339 (5)	
H13A	0.5386	0.5660	0.6300	0.041*	
C15	0.61098 (18)	0.49624 (12)	0.90861 (17)	0.0315 (4)	
H15A	0.6676	0.5090	0.9916	0.038*	
C14	0.61647 (18)	0.54999 (11)	0.81580 (18)	0.0332 (5)	
H14A	0.6755	0.5997	0.8359	0.040*	
C23	−0.36913 (18)	0.27314 (12)	1.15025 (16)	0.0290 (4)	
H23A	−0.3515	0.3259	1.2000	0.044*	
H23B	−0.3622	0.2223	1.2016	0.044*	
H23C	−0.4635	0.2759	1.0848	0.044*	

### Atomic displacement parameters (Å^2^)

**Table:**

	*U*^11^	*U*^22^	*U*^33^	*U*^12^	*U*^13^	*U*^23^
O1	0.0239 (6)	0.0211 (6)	0.0327 (7)	0.0069 (5)	0.0122 (5)	0.0020 (5)
N1	0.0175 (7)	0.0178 (7)	0.0251 (7)	0.0009 (5)	0.0115 (6)	0.0001 (5)
N2	0.0186 (7)	0.0179 (7)	0.0218 (7)	−0.0012 (5)	0.0077 (6)	−0.0007 (6)
C18	0.0239 (9)	0.0178 (8)	0.0236 (9)	−0.0010 (7)	0.0084 (7)	−0.0019 (7)
C19	0.0185 (8)	0.0245 (9)	0.0268 (9)	−0.0041 (7)	0.0076 (7)	0.0006 (7)
C6	0.0198 (8)	0.0183 (8)	0.0271 (9)	0.0023 (7)	0.0094 (7)	−0.0022 (7)
C8	0.0205 (8)	0.0195 (8)	0.0231 (8)	−0.0037 (7)	0.0108 (7)	−0.0019 (7)
C9	0.0169 (8)	0.0146 (8)	0.0224 (8)	−0.0011 (6)	0.0080 (7)	−0.0033 (6)
C11	0.0163 (8)	0.0205 (8)	0.0259 (9)	0.0039 (6)	0.0123 (7)	0.0011 (7)
C22	0.0214 (9)	0.0208 (9)	0.0255 (9)	−0.0043 (7)	0.0091 (7)	−0.0014 (7)
C5	0.0195 (8)	0.0198 (8)	0.0241 (9)	−0.0014 (7)	0.0112 (7)	−0.0015 (7)
C10	0.0207 (9)	0.0257 (9)	0.0277 (9)	0.0017 (7)	0.0140 (7)	−0.0037 (7)
C17	0.0194 (8)	0.0183 (8)	0.0188 (8)	0.0027 (6)	0.0067 (7)	0.0041 (6)
C4	0.0172 (8)	0.0158 (8)	0.0201 (8)	−0.0019 (6)	0.0071 (7)	−0.0020 (6)
C20	0.0189 (8)	0.0239 (9)	0.0192 (8)	0.0017 (7)	0.0065 (7)	0.0027 (7)
C7	0.0240 (9)	0.0161 (8)	0.0221 (9)	−0.0008 (7)	0.0086 (7)	0.0002 (6)
C21	0.0250 (9)	0.0242 (9)	0.0224 (9)	−0.0011 (7)	0.0102 (7)	−0.0043 (7)
C3	0.0161 (8)	0.0153 (8)	0.0215 (8)	−0.0018 (6)	0.0057 (7)	−0.0027 (6)
C2	0.0187 (8)	0.0181 (8)	0.0218 (8)	−0.0018 (7)	0.0078 (7)	−0.0036 (7)
C16	0.0245 (9)	0.0267 (9)	0.0266 (9)	0.0012 (7)	0.0136 (8)	0.0030 (7)
C12	0.0179 (8)	0.0421 (11)	0.0277 (9)	0.0035 (8)	0.0102 (7)	0.0101 (8)
C13	0.0218 (9)	0.0368 (11)	0.0476 (12)	0.0062 (8)	0.0185 (9)	0.0211 (9)
C15	0.0265 (10)	0.0343 (10)	0.0362 (11)	−0.0052 (8)	0.0152 (9)	−0.0097 (8)
C14	0.0229 (9)	0.0217 (9)	0.0617 (13)	−0.0011 (7)	0.0238 (9)	−0.0011 (9)
C23	0.0250 (9)	0.0378 (10)	0.0264 (9)	−0.0001 (8)	0.0125 (8)	0.0007 (8)

### Geometric parameters (Å, º)

**Table:**

O1—C2	1.2160 (18)	C22—H22A	0.9300
N1—C2	1.3690 (19)	C5—C4	1.393 (2)
N1—C9	1.4137 (19)	C5—H5A	0.9300
N1—C10	1.4636 (19)	C10—H10A	0.9700
N2—C3	1.2767 (19)	C10—H10B	0.9700
N2—C17	1.4210 (19)	C4—C3	1.469 (2)
C18—C19	1.381 (2)	C20—C21	1.389 (2)
C18—C17	1.392 (2)	C20—C23	1.508 (2)
C18—H18A	0.9300	C7—H7A	0.9300
C19—C20	1.388 (2)	C21—H21A	0.9300
C19—H19A	0.9300	C3—C2	1.534 (2)
C6—C5	1.389 (2)	C16—C15	1.385 (2)
C6—C7	1.389 (2)	C16—H16A	0.9300
C6—H6A	0.9300	C12—C13	1.383 (2)
C8—C9	1.379 (2)	C12—H12A	0.9300
C8—C7	1.393 (2)	C13—C14	1.379 (3)
C8—H8A	0.9300	C13—H13A	0.9300
C9—C4	1.404 (2)	C15—C14	1.375 (2)
C11—C16	1.385 (2)	C15—H15A	0.9300
C11—C12	1.389 (2)	C14—H14A	0.9300
C11—C10	1.511 (2)	C23—H23A	0.9600
C22—C21	1.388 (2)	C23—H23B	0.9600
C22—C17	1.394 (2)	C23—H23C	0.9600
			
C2—N1—C9	110.69 (13)	C5—C4—C3	133.73 (14)
C2—N1—C10	124.27 (13)	C9—C4—C3	106.64 (13)
C9—N1—C10	124.73 (13)	C19—C20—C21	117.63 (15)
C3—N2—C17	123.20 (13)	C19—C20—C23	120.58 (15)
C19—C18—C17	120.45 (15)	C21—C20—C23	121.78 (15)
C19—C18—H18A	119.8	C6—C7—C8	121.26 (15)
C17—C18—H18A	119.8	C6—C7—H7A	119.4
C18—C19—C20	121.47 (15)	C8—C7—H7A	119.4
C18—C19—H19A	119.3	C22—C21—C20	121.84 (15)
C20—C19—H19A	119.3	C22—C21—H21A	119.1
C5—C6—C7	120.88 (15)	C20—C21—H21A	119.1
C5—C6—H6A	119.6	N2—C3—C4	136.53 (14)
C7—C6—H6A	119.6	N2—C3—C2	117.71 (13)
C9—C8—C7	117.45 (14)	C4—C3—C2	105.65 (12)
C9—C8—H8A	121.3	O1—C2—N1	126.74 (15)
C7—C8—H8A	121.3	O1—C2—C3	126.97 (14)
C8—C9—C4	122.25 (14)	N1—C2—C3	106.29 (13)
C8—C9—N1	127.10 (14)	C15—C16—C11	120.69 (16)
C4—C9—N1	110.64 (13)	C15—C16—H16A	119.7
C16—C11—C12	118.72 (15)	C11—C16—H16A	119.7
C16—C11—C10	120.68 (14)	C13—C12—C11	120.44 (17)
C12—C11—C10	120.60 (15)	C13—C12—H12A	119.8
C21—C22—C17	119.70 (14)	C11—C12—H12A	119.8
C21—C22—H22A	120.1	C14—C13—C12	120.23 (16)
C17—C22—H22A	120.1	C14—C13—H13A	119.9
C6—C5—C4	118.71 (14)	C12—C13—H13A	119.9
C6—C5—H5A	120.6	C14—C15—C16	120.09 (17)
C4—C5—H5A	120.6	C14—C15—H15A	120.0
N1—C10—C11	113.38 (12)	C16—C15—H15A	120.0
N1—C10—H10A	108.9	C15—C14—C13	119.82 (17)
C11—C10—H10A	108.9	C15—C14—H14A	120.1
N1—C10—H10B	108.9	C13—C14—H14A	120.1
C11—C10—H10B	108.9	C20—C23—H23A	109.5
H10A—C10—H10B	107.7	C20—C23—H23B	109.5
C18—C17—C22	118.88 (15)	H23A—C23—H23B	109.5
C18—C17—N2	118.41 (14)	C20—C23—H23C	109.5
C22—C17—N2	122.27 (14)	H23A—C23—H23C	109.5
C5—C4—C9	119.44 (14)	H23B—C23—H23C	109.5
